# Silencing of KIF3B Suppresses Breast Cancer Progression by Regulating EMT and Wnt/*β*-Catenin Signaling

**DOI:** 10.3389/fonc.2020.597464

**Published:** 2021-01-19

**Authors:** Chengqin Wang, Runze Zhang, Xiao Wang, Yan Zheng, Huiqing Jia, Haiyan Li, Jin Wang, Ning Wang, Fenggang Xiang, Yujun Li

**Affiliations:** ^1^ Department of Pathology, The Affiliated Hospital of Qingdao University, Qingdao, China; ^2^ Department of Pathology, School of Basic Medicine, Qingdao University, Qingdao, China; ^3^ Department of Pathology, Affiliated Yantai Yuhuangding Hospital, Qingdao University, Qingdao, China

**Keywords:** kinesin family member 3B, breast cancer, progression, glycogen synthase kinase 3*β*, Wnt/*β*-catenin signaling, epithelial mesenchymal transition, Dvl2

## Abstract

Breast cancer is the most common malignant tumors in women. Kinesin family member 3B (KIF3B) is a critical regulator in mitotic progression. The objective of this study was to explore the expression, regulation, and mechanism of KIF3B in 103 cases of breast cancer tissues, 35 metastatic lymph nodes and breast cancer cell lines, including MDA-MB-231, MDA-MB-453, T47D, and MCF-7. The results showed that KIF3B expression was up-regulated in breast cancer tissues and cell lines, and the expression level was correlated with tumor recurrence and lymph node metastasis, while knockdown of KIF3B suppressed cell proliferation, migration, and invasion both *in vivo* and *in vitro*. In addition, UALCAN analysis showed that KIF3B expression in breast cancer is increased, and the high expression of KIF3B in breast cancer is associated with poor prognosis. Furthermore, we found that silencing of KIF3B decreased the expression of Dvl2, phospho-GSK-3*β*, total and nucleus *β*-catenin, then subsequent down-regulation of Wnt/*β*-catenin signaling target genes such as CyclinD1, C-myc, MMP-2, MMP-7 and MMP-9 in breast cancer cells. In addition, KIF3B depletion inhibited epithelial mesenchymal transition (EMT) in breast cancer cells. Taken together, our results revealed that KIF3B is up-regulated in breast cancer which is potentially involved in breast cancer progression and metastasis. Silencing KIF3B might suppress the Wnt/*β*-catenin signaling pathway and EMT in breast cancer cells.

## Introduction

Breast cancer is the most common cancer and the leading cause of death in women around the world (1). Over the past decades, therapeutic management, including surgical resection in combination with hormonal therapy, chemo-radiotherapy and biological therapy, has achieved great progress for this disease. However, the survival of advanced-stage breast cancer is still very poor (2). Moreover, the efficiency of the currently available chemotherapy is limited and the development of drug resistance among breast cancer cells further complicates the problem (3). Therefore, the detection of novel therapeutic targets may provide better treatment to such breast cancer patients.

It has been known that Wnt signaling pathway plays a critical role in cell proliferation, differentiation, and migration (4). Dvl2 (Disheveled 2), GSK-3*β* and *β*-catenin are the main regulators in Wnt signaling (5, 6). During Wnt/*β*-catenin pathway activation, Dvl2 protein is up-regulated and GSK-3*β* is inhibited by phosphorylation at the Ser9 site, which decreased *β*-catenin degradation and led to *β*-catenin accumulating in the cytosol. *β*-catenin then travels to the nucleus and interacts with the T-cell factor/lymphoid enhancer factor (TCF/LEF) to activate specific Wnt target genes, including C-myc, CyclinD1, MMP-2, MMP-7 and MMP-9, resulting in tumorigenesis and metastasis (7–10). Moreover, Wnt/*β*-catenin signaling is also associated with the regulation of epithelial cell phenotype and the maintenance of tissue homeostasis. Impaired Wnt signaling pathway could contribute to epithelial mesenchymal transition (EMT) (11). It has also been shown that EMT plays a vital role in tumor progression and metastasis (12–14). EMT is a crucial event whereby epithelial cells lose their epithelial characteristics and acquire mesenchymal phenotype (15). At the molecular level, it’s manifested with reduced E-cadherin expression but increased mesenchymal marker (Vimentin) and transcription factors’ (Slug and Snail) expression (16–18). During EMT, epithelial cells lose cell junction and cell polarity, becoming isolated and motile (19, 20). The significance of EMT and Wnt/*β*-catenin signaling in breast cancer proliferation and metastasis has been reported in previous studies (21–23).

Kinesin super family proteins (KIFs) were first discovered in the 1980s. As a class of conserved microtubule-dependent molecular motor proteins, KIFs transport intracellular cargo along microtubules (24, 25). KIFs are also involved in multiple cellular processes, such as mitosis, meiosis, and vesicle transport. Mitosis is a complex and highly controlled process of cell division. Any abnormality during mitosis can lead to cell death, gene deletion, chromosome translocation, duplication, and even carcinogenesis (26, 27). KIF3 is a subfamily of KIFs, including KIF3A/3B heterodimer and kinesin-associated protein KAP3 (28, 29). This complex is involved in the intracellular transport of membrane-bound complexes and organelles in epithelial cells, neurons and other cells (30–32). KIF3B consists of a group of molecular motors and function in vesicle transport, spermatogenesis, mitotic progression, and intravasation of cancer cells for metastasis (33). Recently, the role of KIF3B in cancer progression has been widely studied. KIF3B has been shown to be over-expressed in multiple human cancers, such as gastric cancer, oral squamous cell carcinoma, pancreatic cancer, prostate cancer, seminoma, hepatocellular carcinoma, and acute lymphoblastic leukemia (34–40). Silencing of KIF3B in an avian embryo model significantly inhibited vasculotropism and metastasis in prostate cancer cell PC3 and other cancer cells (33). Increased expression of KIF3B was correlated with poor survival in patients with hepatocellular carcinoma while its inhibition decreased cancer growth and induced tumor apoptosis (39). Therefore, KIF3B is a novel therapeutic target to block cancer metastasis and inhibit cancer development. However, the role of KIF3B in breast cancer with Wnt signaling and the EMT process in breast cancer remains subtle.

In this study, we studied the expression level of KIF3B in breast cancer tissues and metastatic lymph nodes and its association with cancer progression. We also explored the role of KIF3B in EMT and in regulation of Wnt/*β*-catenin signaling pathway and identified the downstream targets CyclinD1, C-myc, MMP-2, MMP-7 and MMP-9 *in vitro* and *in vivo*. Our results indicate that silencing of KIF3B could suppress breast cancer progression by regulating Wnt/*β*-catenin signaling and EMT, providing support that KIF3B could serve as a potential therapeutic target for the treatment of breast cancer.

## Methods

### Database Searching for Gene Expression and Survival Analysis

UALCAN (http://ualcan.path.uab.edu/) is an interactive web resource for analyzing transcriptome data of cancers from TCGA database. Using UALCAN, we evaluated the mRNA expression level of KIF3B in breast cancer from various angles such as sample types (normal/primary tumor), cancer stages (stages 1, 2, 3, and 4), and nodal metastasis status (N0, 1, 2, 3, and 4). Furthermore, we used UALCAN to analyze the relationship between KIF3B expression and the survival of breast cancer patients. Samples were categorized into two groups: High expression with TPM (Transcripts Per Million) values above upper quartile and Low/Medium expression with TPM (Transcripts Per Million) below upper quartile (41).

### Patients and Tissues

103 breast cancer tissues, corresponding adjacent normal tissues, 35 metastatic lymph nodes and related clinical information (**Table 1**) were obtained from the Affiliated Hospital of Qingdao University during 2016 and 2017. Specimens from cancer tissue and adjacent normal tissue were obtained during the surgery and were immediately dipped in 10% formalin for immunohistochemistry (IHC) analysis. Nine patients had both cancer and adjacent tissue sufficiently large so that a portion of the tissue was immediately frozen in liquid nitrogen and stored for RNA/protein analysis. The study was approved by the ethics committee of the Affiliated Hospital of Qingdao University.

**Table 1:** Association between KIF3B in breast cancer and patient characteristics.

Variables	n	Mean ± SD	*P* value
Age			
≤60 years	55	5.710 ± 0.658	0.270
>60 years	48	5.542 ± 0.743	
Tumor size (cm)			
≤2	41	5.659 ± 0.728	0.706
>2	62	5.613 ± 0.686	
Tumor grade			
II	62	5.661 ± 0.745	0.072
III	41	5.585 ± 0.631	
Carcinoma	103	5.631 ± 0.700	<0.001*
Adjacent tissues	103	4.505 ± 0.640	
Lymph node metastasis			
Negative	68	5.427 ± 0.676	<0.001*
Positive	35	6.029 ± 0.568	
Vascular invasion			
Presence	12	6.083 ± 0.515	0.317
Absence	91	5.571 ± 0.701	
Primary tumor	35	6.029 ± 0.568	<0.001*
Metastatic tumor in lymph nodes	35	6.800 ± 0.677	
Tumor recurrence			
Positive	30	6.067 ± 0.583	0.008*
Negative	73	5.452 ± 0.668	

*Significant at <0.05.

### Real-Time RT-PCR Analysis

Total RNA of fresh tissues was isolated using RNAiso reagent (Takara Bio, Japan) according to the manufacturer’s instructions. RT-PCR was performed as described previously (42). Forward and reverse primer sequences for KIF3B and GAPDH were as follows, respectively: KIF3B-F: GATGTTAAGCTGGGGCAGGT, KIF3B-R: TTTGCCGTCCACTAGAGCAG. GADPH-F: ACCACAGTCCATGCCATCAC, GADPH-R: TCCACCACCCTGTTGCTGTA.

### Western Blot Analysis

Total proteins were extracted from fresh tissues and cultured cells using RIPA lysis buffer containing protease inhibitor PMFS. Nuclear proteins from cells were prepared according to the manufacturer’s instructions of Nuclear Protein Extraction Kit (Solarbio, China). Western blot was performed as described previously (42). Antibodies used in the study were as follows: KIF3B and Vimentin (Santa Cruz Biotechnology, all at dilution 1:1,000), GAPDH, *β*-catenin, GSK-3*β*, p-GSK-3*β* ^ser9^, Cyclin D1, C-myc, E-cadherin, MMP-2, MMP-9 (Abcam, all at dilution 1:1,000), Dvl2, MMP-7, Slug and Snail (Bioss, all at dilution 1:1,000), *β*-actin, Histone H3 (TransGen Biotech, all at dilution 1:4,000).

### Immunohistochemistry Analysis

Briefly, after deparaffinization and hydration, the slides were inactivated endogenous peroxidase by using 3% hydrogen peroxide and heat-pretreated in ethylene diamine tetraacetic acid (PH 8.0) by a microwave oven for 5 min. Then, anti-KIF3B (Abcam, USA, ab152976, 37°C, 2h, 1:200) and secondary antibody (37°C, 30 min) were incubated. Sections were stained with Diaminobenzidine (DAB) and counterstained with hematoxylin. PBS was used as negative control. Other antibodies were as follows: *β*-catenin, CyclinD1, C-myc, p-GSK-3*β* ^ser9^, E-cad, Vimentin, MMP-2 and MMP-9 (Bioworld Technology, all at dilution 1:200), Dvl2, MMP-7, Slug and Snail (Bioss, all at dilution 1:200). Independent Histologic and IHC evaluation were conducted by two pathologists. IHC staining of KIF3B was scored as described previously (43, 44). The scores of staining intensity (0, none; 1, weak; 2, intermediate; and 3, strong) and positive tumor cell proportion (0, none; 1, <1/100; 2, 1/100 to 1/10; 3, 1/10 to 1/3; 4, 1/3 to 2/3; and 5, > 2/3) were summed up to obtain a total score (ranging from 0 to 8).

### Cell Culture

Human breast cancer cell lines, MDA-MB-231, MDA-MB-453, T47D, and MCF-7 were purchased from the American Type Culture Collection (ATCC, VA, USA) and cultured in Dulbecco’s modified eagle medium (Hyclone, Logan, UT, USA) with 10% fetal bovine serum at 37°C and 5% CO_2_.

### Lentiviral Transduction

Lentiviral production was synthesized by Hanbio (Shanghai, China). The sequences of KIF3B-shRNA and scramble controls were as follows (38):

KIF3B-siRNA1#, sense: 5′-GCAGAAACGUCGAGAAAGATT-3′; and antisense: 5′ -UCUUUCUC GACGUUUCUGCTT-3′;

KIF3B-siRNA2#, sense = 5′-GAUCCCAGAAUCAACAAUATT-3′; and antisense = 5′-UAUUGUUGAUUCUGGGAUCTT-30′;

KIF3B-siRNA3#, sense: 5′-GGAGCUGAAACUCAAGCAUTT-3′; and antisense =

5′-AUGCUUGAGUUUCAGCUCCTT-3′;

negative control (Scr-shRNA):sense = 5′-UUCUCCGAACGUGUCACGUdTdT-3′; and antisense = 5′-ACGUGACACGUUCGGAGAAdTdT-3′. The lentiviral transduction was performed according to the manufacturer’s instructions, then we observed the transfection efficiency with fluorescence, and perform re-transfection if the fluorescence becomes weak, without screening of cell lines stably expressing KIF3B-shRNA. Over-expression of KIF3B was performed by over-expression vector plasmid (KIF3B). Empty plasmids were used as negative control (NC). Plasmids were purchased from Genechem (Genechem, China), and all constructs were confirmed by sequencing.

### Cell Proliferation Assays

The procedure of MTT assay has been described previously (42). For colony formation assays, 1 × 10^4^ cells were added to 1 ml of the growth medium with 0.3% agar after transfection with KIF3B shRNA lentivirus then layered onto 2 ml of 0.6% agar beds in 6-well culture plate. After three weeks of culture, cells were then stained with crystal violet (0.05%) for 30 min and photographed. Colonies were counted using Image-ProPlus 6.0 software (Media Cybernatics, Inc.,Bethesda, USA).

### Cell Cycle Analysis

Cells were fixed in 70% ethanol for 1 h at 4°C overnight and then incubated with 1 mg/ml RNaseA for 30 min at 37°C. Cells were stained with propidium iodide (50 μg/ml) in PBS containing 0.5% Tween-20 and analyzed using flow cytometry (BD Accuri C6, USA).

### Transwell Migration and Invasion Assays

Transwell chambers (Corning, 8 um poly carbonate membrane) were used according to the manufacturer’s protocol. Briefly, 600 ul of medium containing 15% FBS was added into the lower chamber. 4 × 10^4^ cells (for migration) were plated in the upper chamber containing 200 ul of serum-free medium. After 24 h, the cells in the upper chamber were removed with a cotton swab. 8 × 10^5^ cells (for invasion) were added to the upper chamber with Matrigel (Corning, 1:8). After 48 h, the cells in the upper chamber were removed with a cotton swab. The migratory and invasive cells on the lower filters were fixed with methanol and then stained with Giemsa. Cells in five random view fields were counted under the microscope.

### Tumor Growth and Metastasis Assays in Nude Mice

All studies using animal were approved by the Animal Ethics Committee of Qingdao University, China. In short, 4 × 10^6^ Scr-shRNA and KIF3B-shRNA MDA-MB-231 cells were subcutaneously implanted into BABL/c nude mice with five mice in each group. Size and weight of tumors were recorded every 7 days, and the tumor volume was measured using the following formula: volume (mm^3^) = (width^2^ × length)/2. Twenty BABL/c mice were randomly divided into two groups (10 in each group) and injected with Scr-shRNA and KIF3B-shRNA MDA-MB-231 cells (2 × 10^6^) *via* tail vein. Six weeks later, mice were euthanized. The lungs were stained with HE. The whole lung tissue of each mouse was sectioned, and metastatic nodules were counted in high-power fields under a microscope.

### Statistical Analysis

All statistical analyses were performed using SPSS 23.0 software. All values were presented as mean ± SD. Wilcoxon’s test was used for non-normal distributed data. The Student’s t test was used for data that were normally distributed. Differences were considered statistically significant at *P* < 0.05 and *P* < 0.01.

## Results

### Bioinformatic Analysis of KIF3B Expression and Prognostic Value in Breast Cancer Patients

**Figure 1:**
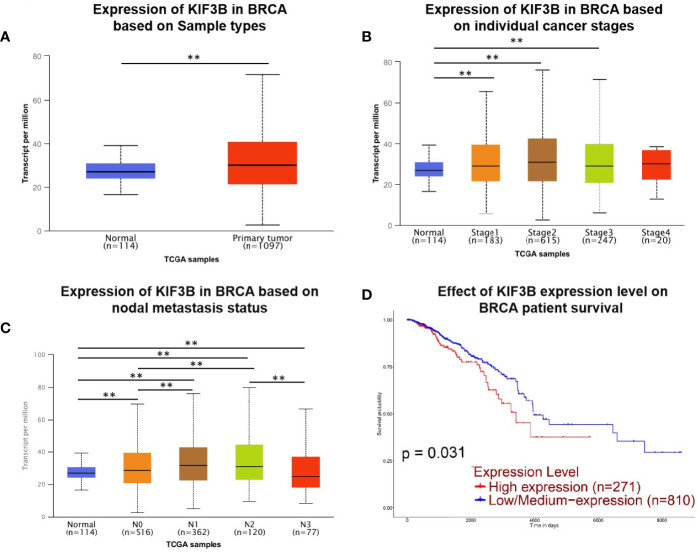
UALCAN gene analysis of breast cancer samples from the TCGA database. **(A–C)** Comparison of KIF3B mRNA expression between normal and breast cancer samples, individual cancer stages and nodal metastasis status. **(D)** Kaplan–Meier survival curves of patients with high and low/medium expression of KIF3B in breast cancer (**P < 0.01).

We evaluated KIF3B expression and prognostic value in breast cancer patients by using the UALCAN database. The results showed that KIF3B is over-expressed in primary breast tumors (n = 1097) compared to normal tissues (n = 114) (**Figure 1A**, *P* < 0.01). We observed that KIF3B expression increased in breast cancer stages 1–3 compared to normal samples (**Figure 1B**, *P* < 0.01). The expression of KIF3B is increased in breast cancer N0–N2 status, but decreased in N3 status (**Figure 1C**, *P* < 0.01). Furthermore, breast cancer patients with high-expression of KIF3B (n = 271) displayed worse survival compared to the patients with low/medium-expression of KIF3B (n = 810) (**Figure 1D**, *P* < 0.01).

### Over-Expression of KIF3B in Breast Cancer

**Figure 2:**
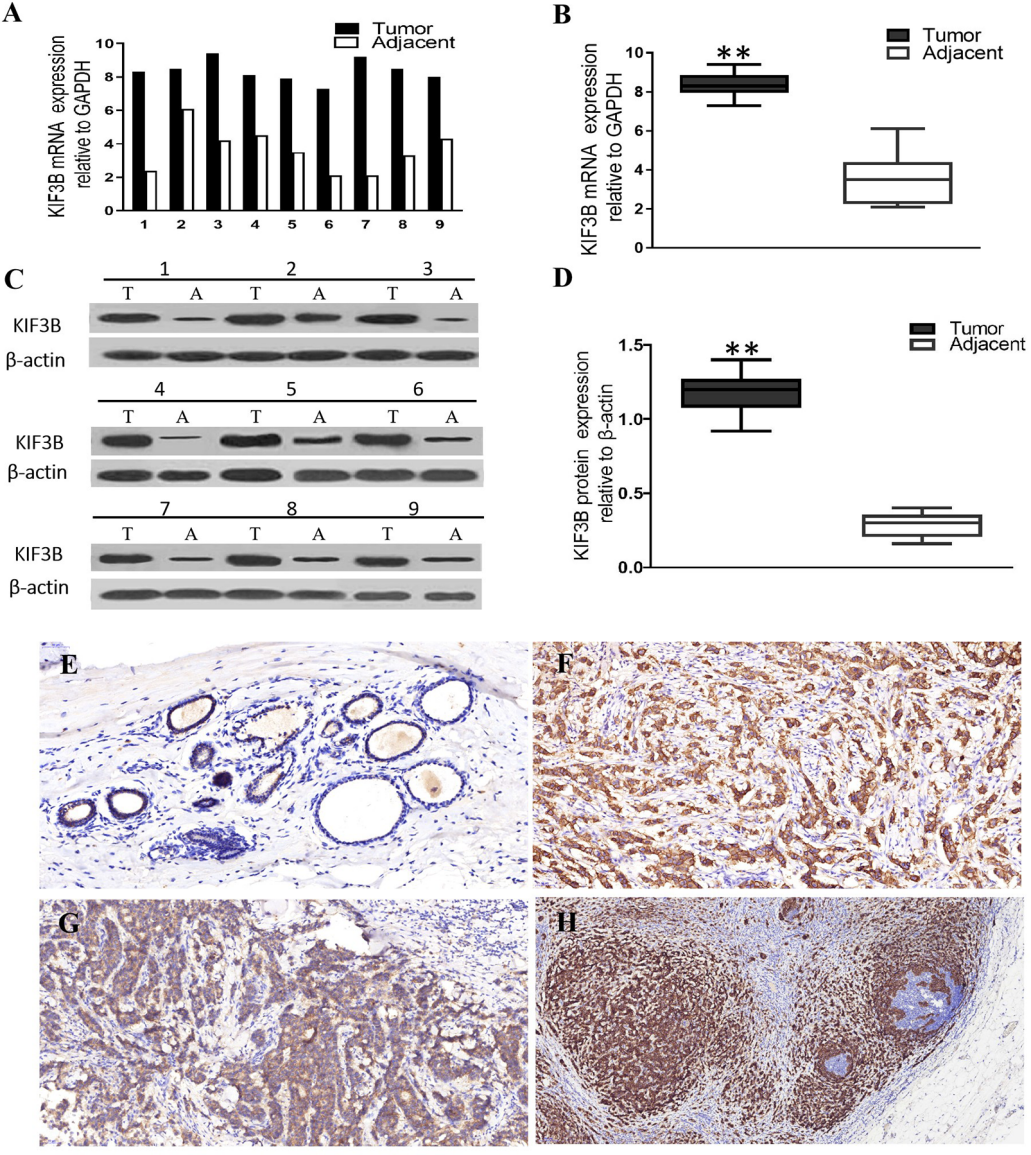
KIF3B expressions were higher in breast cancer than in adjacent tissues at both mRNA and protein levels. **(A, B)** Real time RT-PCR. **(C, D)** Western blot. T, tumor; A, adjacent tissue. GAPDH and *β*-actin were used as control (**P < 0.01). **(E, F)** Immunohistochemistry showing over-expression of KIF3B in cancer cells than in corresponding adjacent tissues, and in cancer cells metastasizing to lymph node than in the corresponding primary foci. **(E)** Adjacent tissue, **(F)** breast cancer, **(G)** primary foci, **(H)** lymph node metastasis. DAB (brown) served as chromogen. (**E**: ×200, **F**: ×200, **G**: ×400, **H**: ×100).

Real time RT-PCR and western blot assays were performed to examine the expression level of KIF3B in breast cancer tissues and nine pairs of fresh tissues. The results showed that both the mRNA (**Figures 2A, B**, *P* < 0.01) and protein levels (**Figures 2C, D**, *P* < 0.01) of KIF3B in the tumor tissues were markedly higher than that of the corresponding adjacent tissues, demonstrating that KIF3B was up-regulated in breast cancer. We further detected KIF3B expression by immunohistochemistry and confirmed that KIF3B expression was higher in breast cancer than in corresponding adjacent tissues (*P* < 0.01) (**Figures 2E, F**; **Table 1**).

### Relationship Between KIF3B Expression and Clinicopathological Factors of Breast Cancer Patients

By analyzing the relationship between the expression of KIF3B and the clinicopathological features of the patients, we found that KIF3B expression in lymph node metastasis was significantly higher than in primary focus (*P* < 0.01) (**Figures 2G, H**, **Table 1**). Also, we found that the expression of KIF3B in the primary tumors with lymph node metastasis was higher than those without lymph node metastasis (**Table 1**, *P* < 0.01). Moreover, patients with recurrent carcinoma showed higher expression of KIF3B than those without recurrence (**Table 1**, *P* < 0.01). No significant differences in age, tumor size, grade or vascular invasion were observed (**Table 1**).

### Effect of KIF3B Knockdown in Breast Cancer Cell Lines

**Figure 3:**
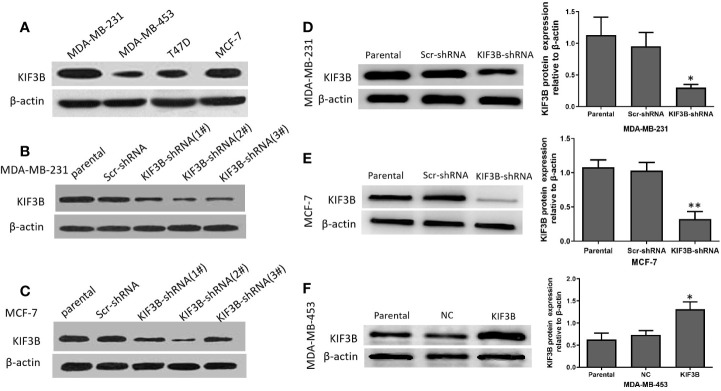
The silencing and over-expression efficiency of KIF3B in breast cancer cell lines. **(A)** Western blot analysis of KIF3B protein expression in MDA-MB-231, MDA-MB-453, T47D and MCF-7 cells. **(B, C)** The knockdown efficiency of KIF3B-shRNA (1#, 2# and 3#) in MDA-MB-231 and MCF-7 cells. The KIF3B-shRNA 2# was selected in the following experiment because of its highest efficiency in silencing of KIF3B. **(D, E)** KIF3B was significantly down-regulated in MDA-MB-453 and MCF-7 cells. **(F)** MDA-MB-453 cells were used to over-express KIF3B by KIF3B expression plasmid, and Western blot analysis showed the expression of KIF3B peaked at 48h after KIF3B transfection (*P < 0.05; **P < 0.01). β-actin was used as the control. Scr-shRNA or NC was used as a negative control. Data were expressed as the gray-scale ratio of KIF3B protein relative to that of β-actin. All data were presented as mean ± SD. All the experiments were repeated three times.

The KIF3B expression in MDA-MB-231, MDA-MB-453, T47D, and MCF-7 cell lines was examined using western blot analyses (**Figure 3A**). Because of high expression of KIF3B, MDA-MB-231, and MCF-7, cells were used for knockdown KIF3B gene by KIF3B-shRNA1#, 2# and 3#. The KIF3B-shRNA 2# was shown to be most effective (**Figures 3B, C**) and so was chosen for silencing of KIF3B gene. After transfection with KIF3B-shRNA 2#, the KIF3B protein level was significantly decreased in MDA-MB-231and MCF-7 cells (**Figures 3D, E**). Meanwhile, due to the lowest expression of KIF3B (**Figure 3A**), MDA-MB-453 cell line was used for over-expressing KIF3B (**Figure 3F**).

### Silencing of KIF3B Suppresses Cell Proliferation and Induces G2/M Arrest

**Figure 4:**
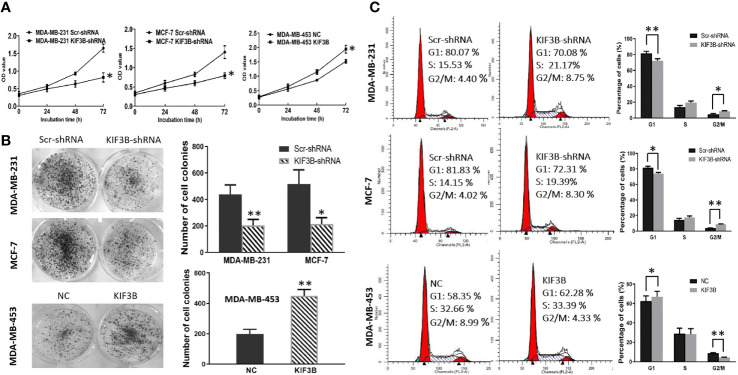
Silencing of KIF3B inhibits tumor cell proliferation and induces G2/M arrest. **(A)** Cell growth was measured by the MTT assay. Absorbance was measured at 490 nm. The data were presented as the means of six separated experiments, each performed in triplicate. **(B)** Colony formation of KIF3B knockdown and over-expression were photographed and colony numbers were illustrated in histogram. **(C)** Flow cytometry revealed the distribution of cell phase in the breast cancer cell lines. Data were shown as mean ± SD from three independent experiments (*P < 0.05; **P < 0.01).

MTT assay and colony-formation assay were performed to explore the effect of KIF3B depletion on cell proliferation. The results showed that the growth rate of the KIF3B-shRNA group was markedly slower than the Scr-shRNA group in MDA-MB-231 and MCF-7 cells. And the growth rate of KIF3B over-expression group was significantly faster than that of the NC group in MDA-MB-453 cells (**Figure 4A**, *P* < 0.05). The colony numbers were 438.33 ± 72.14 *vs*. 203.33 ± 46.65 in MDA-MB-231 cells (*P* < 0.01) and 516.33 ± 107.15 *vs*. 214.67 ± 47.98 in MCF-7 cells, respectively, before and after KIF3B knockdown (**Figure 4B**, *P* < 0.05). The colony numbers were 198.33 ± 31.01 *vs*. 450.00 ± 41.73 in MDA-MB-453 cells (*P* < 0.01) before and after KIF3B over-expression (**Figure 4B**). These findings indicate that KIF3B depletion could inhibit cell growth.

Flow cytometry was used to further investigate the effect of KIF3B on cell cycle. The cell phase distribution of Scr-shRNA and KIF3B-shRNA MDA-MB-231 cells was as follows (**Figure 4C**): G1 phase: 81.53 ± 2.66% *vs*. 72.27 ± 2.60% (*P* < 0.01); S phase: 13.85 ± 1.99% *vs*. 19.33 ± 2.00%; G2/M phase: 4.62 ± 1.01% *vs*. 8.40 ± 0.65% (*P* < 0.05). The cell phase distribution of Scr-shRNA and KIF3B-shRNA MCF-7 cells was as follows: G1 phase: 81.78 ± 1.81% *vs*. 73.93 ±1.65% (*P* < 0.05); S phase: 14.41 ± 1.80% *vs.* 17.41 ± 2.02%; G2/M phase: 3.81 ± 0.18% *vs.* 8.65 ± 0.38% (*P* < 0.01). Compared with the Scr-shRNA group, the proportion of G1 phase was significantly decreased and G2/M phase was markedly increased in KIF3B-shRNA group, indicating a significant G2/M arrest. To further confirm these findings, cell cycle analysis was performed in KIF3B over-expression MDA-MB-453 cells. The cell phase distribution was as follows: G1 phase: 62.47 ± 5.40% *vs*. 67.19 ± 5.35% (*P* < 0.05); S phase: 28.89 ± 5.74% *vs*. 28.44 ± 5.56%; G2/M phase: 8.64 ± 0.73% *vs*. 4.38 ± 0.28% (*P* < 0.01).

### Depletion of KIF3B Suppresses Cell Migration and Invasion Through Inhibiting EMT

**Figure 5:**
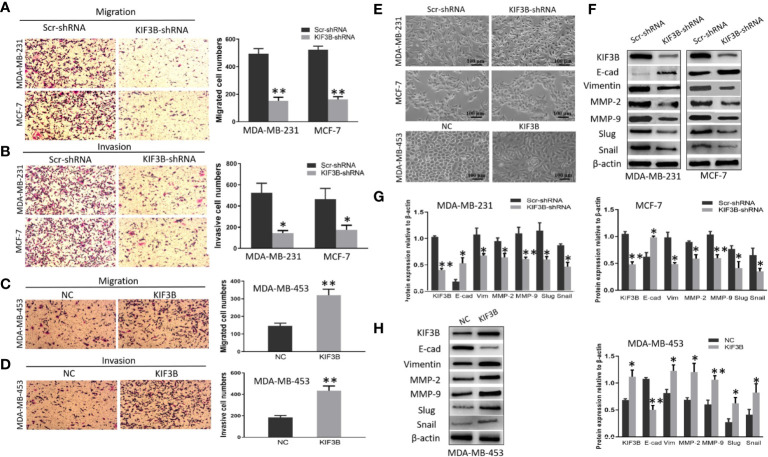
KIF3B knockdown inhibits cell migration and invasion through suppressing EMT. **(A, B)** Transwell and invasion assays showed migration and invasion of MDA-MB-231 and MCF-7 cells in the Scr-shRNA and KIF3B-shRNA groups. **(C, D)** Migration and invasion assay of MDA-MB-453 cells in NC and KIF3B groups. **(E)** The morphology of cells in the Scr-shRNA and KIF3B-shRNA groups (upper) and the NC and KIF3B groups (lower). **(F–H)** Western blot of KIF3B, E-cad, Vimentin, MMP-2, MMP-9, Slug and Snail. All data were mean ± SD values from three independent experiments, each performed in triplicates (*P < 0.05, **P < 0.01).

Transwell assay was conducted to analyze the migration and invasion of MDA-MB-231, MCF-7 and MDA-MB-453 cells. The results showed that the numbers of migrated MDA-MB-231 cells were 494.33 ± 37.69 and 151.00 ± 27.22 in the Scr-shRNA and KIF3B-shRNA groups, respectively (**Figure 5A**, *P* < 0.01). The numbers of migrated MCF-7 cells were 523.33 ± 26.03 and 161.67 ± 20.13 in the Scr-shRNA and KIF3B-shRNA groups, respectively (**Figure 5A**, *P* < 0.01). These data suggested that cell migration was inhibited by KIF3B knockdown in MDA-MB-231 and MCF-7 cells. Moreover, consistent with migration results, invasion assay showed that the invasive ability of the cells was progressively inhibited after KIF3B silencing (**Figure 5B**, *P* < 0.05).

To further confirm these observations, transwell assays were performed in KIF3B over-expression MDA-MB-453 cells. The results showed that the numbers of migrated MDA-MB-453 cells (321.00 ± 33.78 *vs*. 145.67 ± 16.26, *P* < 0.01, **Figure 5C**) and invasive MDA-MB-453 cells (433.67 ± 44.00 *vs*. 184.00 ± 18.08, *P* < 0.01, **Figure 5D**) were significantly increased in the KIF3B group compared to the control group.

We also discovered that silencing of KIF3B may cause morphological changes in the breast cancer cells. MDA-MB-231and MCF-7 became shortened and more adherent to each other due to KIF3B knockdown. However, over-expression of KIF3B in MDA-MB-453 cells led to elongated morphological appearances and mesenchymal-like properties (**Figure 5E**), suggesting that these cells were undergoing EMT.

To further explore the effect of KIFB in EMT, KIF3B, E-cadherin, Vimentin, MMP-2, MMP-9, Slug and Snail were detected by western blot. Compared with the Scr-shRNA group, increased expression of E-cadherin and decreased expressions of Vimentin, MMP-2, MMP-9, Slug and Snail were observed in KIF3B-shRNA MDA-MB-231and MCF-7 cells. This correlation was then confirmed by KIF3B over-expression in MDA-MB-453 cells, in which the expression of E-cadherin was decreased, and the expressions of Vimentin, MMP-2, MMP-9, Slug and Snail were increased (**Figures 5F–H**). These findings suggest that silencing of KIF3B might inhibit cell migration and invasion through suppressing EMT in breast cancer cells.

### Silencing of KIF3B Suppresses Wnt/*β*-Catenin Signaling Pathway in Breast Cancer Cells

**Figure 6:**
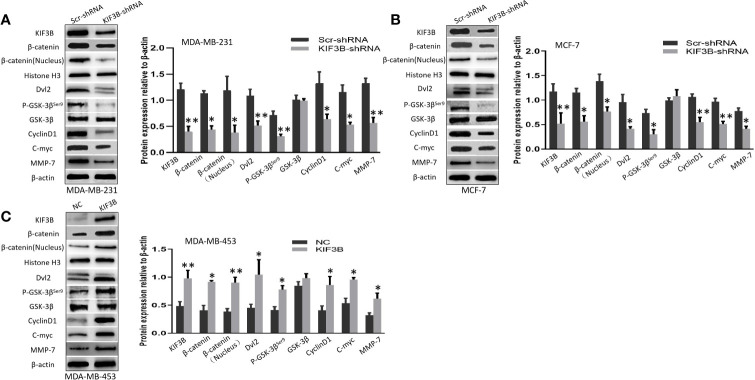
Silencing of KIF3B suppresses Wnt/*β*-catenin signaling pathway in breast cancer cells. **(A–C)** The levels of *β*-catenin, *β*-catenin (Nucleus) (Histone H3 was used as the nucleus control), Dvl2, p-GSK-3*β* ^ser9^, GSK-3*β*, CyclinD1, C-myc, and MMP-7 protein in KIF3B-shRNA MDA-MB-231, MCF-7 cells and KIF3B over-expression MDA-MB-453 cells. All data were presented as mean ± SD. All the experiments were repeated three times (*P < 0.05, **P < 0.01).

To investigate the role of KIF3B on Wnt/*β*-catenin signaling in breast cancer cells, we examined the expression levels of *β*-catenin, *β*-catenin (Nucleus), Dvl2, p-GSK-3*β*, GSK-3*β*, CyclinD1,C-myc and MMP-7 by using western blot in KIF3B-shRNA MDA-MB-231, MCF-7 cells and KIF3B over-expression MDA-MB-453 cells. We found that silencing of KIF3B induced down-regulation of *β*-catenin, *β*-catenin (Nucleus), Dvl2, p-GSK-3β^ser9^, CyclinD1, C-myc and MMP-7 in MDA-MB-231 and MCF-7 cells (**Figures 6A, B**), which were consistent with the results in xenografts. On the contrary, over-expression of KIF3B in MDA-MB-453 cells increased the expression level of *β*-catenin, *β*-catenin (Nucleus), Dvl2, p-GSK-3*β* ^ser9^, CyclinD1, C-myc and MMP-7 (**Figure 6C**). We also examined the effect of KIF3B on the expression of another two target genes of Wnt/*β*-catenin signaling, MMP-2 and MMP-9 (**Figures 5F–H**). All these data indicated that deletion of KIF3B inhibited proliferation and invasion of breast cancer cells probably by regulating the Wnt/*β*-catenin signaling pathway.

### Depletion of KIF3B Suppresses Tumor Growth in Nude Mice

**Figure 7:**
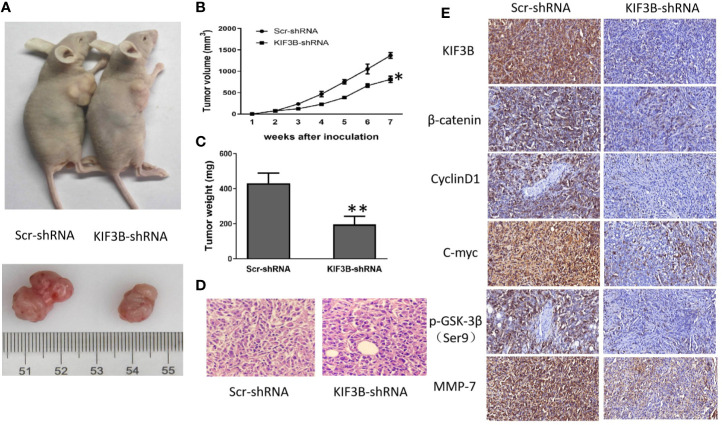
Silencing of KIF3B inhibits tumor growth in nude mice. **(A)** Photograph of Scr-shRNA and KIF3B-shRNA MDA-MB-231 cell xenograft tumors. **(B, C)** Tumor growth curve and average weight were recorded and calculated (*P < 0.05, **P < 0.01). **(D)** Hematoxylin and eosin (HE) staining of the xenografts (left: Scr-shRNA group ×400; right: KIF3B-shRNA group ×400). **(E)** IHC staining of the tumor from the KIF3B-shRNA group and control group (×400).

We next explored the tumor-forming capacity of KIF3B-silencing MDA-MB-231 cells in nude mice. The results showed that the tumor size (812.67 ± 74.78 mm^3^ *vs*. 1370.67 ± 66.01 mm^3^, *P* < 0.05, **Figures 7A, B**) and weight (196.10 ± 46.78 mg *vs*. 431.70 ± 57.78 mg, *P* < 0.01, **Figure 7C**) in KIF3B-shRNA cell xenografts were significantly reduced compared with the Scr-shRNA group; however, hematoxylin and eosin (HE) staining of the xenografts showed no significant difference (**Figure 7D**, left: ×100 Scr-shRNA group ×400; right: KIF3B-shRNA group ×400). Subsequently, the expressions of KIF3B, *β*-catenin, Cyclin D1, C-myc, p-GSK-3*β*ser9, and MMP-7 in xenografts were examined by IHC staining. The results showed down-regulation of KIF3B, *β*-catenin, Cyclin D1, C-myc, p-GSK-3*β* ^ser9^, and MMP-7 in the KIF3B-shRNA group (**Figure 7E**). These findings are consistent with that of the MDA-MB-231 cells (**Figure 6A**), indicating that suppression of KIF3B in MDA-MB-231 cells could inhibit tumor formation and growth.

### Silencing of KIF3B Inhibits Metastasis *In Vivo*

**Figure 8:**
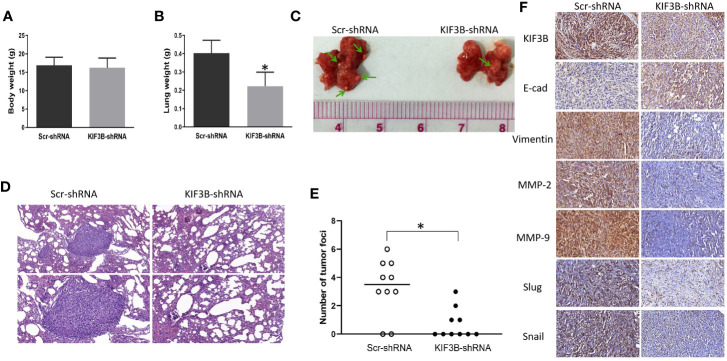
Silencing KIF3B inhibits breast cancer metastasis *in vivo*. Six-week-old female nude BALB/c mice were injected with 1 × 10^6^ Scr-shRNA or KIF3B-shRNA MDA-MB-231 cells *via* the tail vein. Six weeks later, the body weight **(A)** and the lung weight **(B)** of the mice were measured (*P < 0.05). **(C, D)** Images of lung metastasis and HE staining (upper: ×200; lower: ×400). **(E)** The whole lung tissue of each mice was sectioned and metastatic nodules were counted in high-power fields under a microscope (*P < 0.05). **(F)** The expression of KIF3B, E-cad, Vimentin, MMP-2, MMP-9, Slug and Snail in the xenografts (IHC staining, ×400).

To explore the effect of KIF3B on tumor migration and invasion *in vivo*, KIF3B-shRNA MDA-MB-231 cells were intravenously injected into nude mice. There was no significant difference in body weight of the mice after six weeks (**Figure 8A**). However, the weight of the lungs in the KIF3B-shRNA group was significantly less compared with the control group (**Figure 8B**, *P* < 0.05). While eight out of 10 mice presented lung metastasis in the control group, only four out of 10 mice in the KIF3B-shRNA group developed lung metastasis. Furthermore, the number of tumor foci in the KIF3B-shRNA group was much less than that in the control group (**Figures 8C–E**). In addition, the expression level of E-cadherin was up-regulated and expressions of Vimentin, MMP-2, MMP-9, Slug and Snail were reduced in the xenografts in the KIF3B-shRNA group compared with the control group by IHC staining (**Figure 8F**), which were consistent with the results in breast cancer cell lines. Collectively, these results indicate that KIF3B silencing could significantly suppress breast cancer metastasis both *in vivo* and *in vitro*.

## Discussion

Kinesin superfamily proteins (KIFs) play important roles in intracellular transportation and cell division (45). KIF3B, a subunit of KIF3 subfamily proteins, acts as a microtubule-directed motor and is an important regulator in multiple cellular processes, such as mitosis, intracellular transport and cilium assembly (46, 47). In the past few years, a large body of data has shown the association of KIF3B abnormality with tumor proliferation or invasion in several human cancers (34–40). Recently, Li et al. found that KIF3B is highly expressed in breast cancer, and over-expression of tumor-related KIFs correlates with worse outcome of breast cancer patients by bioinformatic analysis (48). However, the expression and related mechanisms of KIF3B in breast cancer have not been experimentally verified.

Our data firstly provided evidence that KIF3B expression was significantly increased at both the mRNA and protein levels in breast cancer tissues compared with the corresponding adjacent tissues. IHC analysis also showed that KIF3B was highly expressed in breast cancer tissues than the adjacent tissues, which confirmed our UALCAN analysis and the result published by Li et al. (48). Furthermore, we found that KIF3B expression in lymph node metastasis was significantly higher than in the primary focus. And the increased expression of KIF3B was correlated with lymph node metastasis and tumor recurrence, suggesting a significant association of high KIF3B expression with tumor growth and metastasis of breast cancer.

Previous study has shown that Wnt/*β*-catenin signaling pathway activation becomes dominant in basal-like breast cancers and predicts poor prognosis, and inhibiting Wnt/*β*-catenin signaling can effectively suppress breast cancer development (22, 23, 49), but the underlying mechanisms are poorly understood. KIF3A,another subunit of the KIF3 subfamily proteins, has been shown to promote proliferation and invasion *via* Wnt signaling in advanced prostate cancer (50). Since KIF3B and KIF3A are similar in sequence and function (28), we investigated whether KIF3B plays a role in regulating Wnt signaling. In this study, we firstly revealed that KIF3B is a potent activator of Wnt signaling in human breast cancer. We found that up-regulation of KIF3B activates the Wnt signaling *via* increasing the expression of Dvl2, p-GSK-3*β* ^ser9^, total and nucleus *β*-catenin, then up-regulation of Wnt signaling target genes such as CyclinD1, C-myc and MMP-7, which are highly expressed in breast cancer (51–54). Meanwhile, silencing of KIF3B could down-regulate CyclinD1 and C-myc and induce cell cycle arrest at G2/M phase. These results indicate the presence of KIF3B/GSK-3*β*/*β*-catenin axis and depletion of KIF3B might be one of the molecular mechanisms inhibiting the Wnt signaling pathway in breast cancer.

EMT could promote breast cancer cell stemness, invasion, and metastasis (49). Wnt/*β*-catenin signaling pathway has been shown to regulate EMT in several types of cancers, including breast cancer (55). In addition, during tumor invasion and metastasis, MMP-2, MMP-7, and MMP-9, the target genes of Wnt/*β*-catenin signaling, function to degrade the ECM and basement membrane so that tumor cells can detach, invade, and metastasize (7, 56). E-cadherin, Vimentin, Slug and Snail were the main factors of EMT (57). In our study, high expression of KIF3B was shown to be associated with lymph node metastasis. Depletion of KIF3B resulted in both morphological changes and suppressed migration and invasion *via* inhibiting EMT in MDA-MB-231 and MCF-7 cells. Furthermore, the up-regulated expression of E-cadherin was accompanied by the down-regulation of Vimentin, MMP-2, MMP-9, MMP-7, Slug and Snail in the KIF3B-shRNA group. These results were further confirmed in the xenografts. From these results, we conclude that depletion of KIF3B might repress migration and invasion of MDA-MB-231 and MCF-7 cells through inhibiting EMT as a result of suppressed Wnt/*β*-catenin signaling.

**Figure 9:**
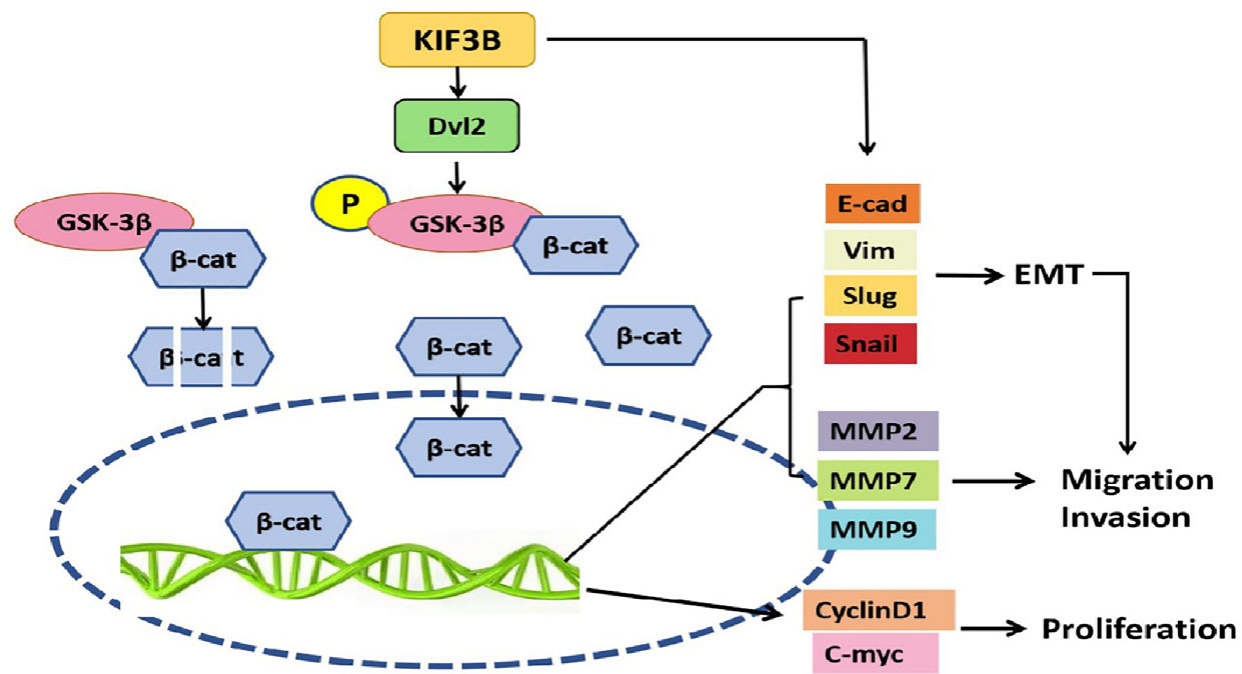
KIF3B might promote proliferation, migration, and invasion through regulating Wnt/*β*-catenin signaling and EMT in breast cancer cells. High expression of KIF3B up-regulates Dvl2 expression and GSK-3*β* phosphorylation, leading to accumulation of *β*-catenin in the nucleus, and subsequently activating the EMT and Wnt target genes transcription, which contributes to the development and progression of breast cancer cells.

Collectively, our results provided support for the first time that KIF3B was highly expressed in breast cancer, and the high level expression was closely associated with lymph node metastasis and tumor recurrence. In addition, KIF3B knockdown might repress proliferation, migration, and invasion through regulating Wnt/*β*-catenin signaling and EMT in breast cancer cells (**Figure 9**), suggesting that KIF3B plays a key regulatory role in cell proliferation and metastasis in breast cancer.

## Data Availability Statement

The raw data supporting the conclusions of this article will be made available by the authors, without undue reservation.

## Ethics Statement

The studies involving human participants were reviewed and approved by the ethics committee of the Affiliated Hospital of Qingdao University. The patients/participants provided their written informed consent to participate in this study. The animal study was reviewed and approved by the Animal Ethics Committee of Qingdao University, China.

## Author Contributions

Conception and design of study: FX, YL. Acquisition of data: CW, RZ, HJ, YZ, JW. Analysis and/or interpretation of data: CW, RZ, HJ, HL, JW, NW. Drafting the manuscript: CW. Revising the manuscript critically for important intellectual content: FX, YL. All authors contributed to the article and approved the submitted version.

## Funding

This research was supported by the Natural Science Foundation of China (Nos.81672606, 81972329, 81702677) and China Postdoctoral Science Foundation (2016M592147).

## Conflict of Interest

The authors declare that the research was conducted in the absence of any commercial or financial relationships that could be construed as a potential conflict of interest.

## Abbreviations

KIF3B, Kinesin family member 3B; RT-PCR, Real Time Polymerase Chain Reaction; shRNA, short hairpin RNA; MTT, 3-(4,5-dimethylthiazol-2-yl)-2,5-diphenyltetrazolium bromide; TCF/LEF, T-cell factor/lymphoid enhancer factor; EMT, Epithelial mesenchymal transition; HCC, Hepatocellular carcinoma; IHC, Immunohistochemistry; DAB, Diaminobenzidine; NC, Negative control; MMP, Matrix metalloproteinase; BRCA, Breast cancer; Dvl2, Disheveled 2; GSK-3*β*, Glycogen synthase kinase 3*β.*
